# Physiological 17β-oestradiol inhibits K_V_7.4 currents via GPER1-dependent PLC/PKC signalling

**DOI:** 10.1080/19336950.2026.2729646

**Published:** 2026-09-10

**Authors:** Elif Karabatak, Skye Lau, Samantha C. Salvage, Thomas A. Jepps, Iain A. Greenwood, Samuel N. Baldwin

**Affiliations:** a School of Health and Medical Sciences, City St George’s, University of London, London, UK; b Vascular Biology Group, Department of Biomedical Sciences, University of Copenhagen, Copenhagen, Denmark

**Keywords:** Kv7.4, GPER1, estradiol, phospholipase C, protein kinase C

## Abstract

K_V_7.4 channels are important regulators of membrane potential and excitability in multiple tissues and are emerging as key effectors of steroid hormone signaling. Here, 17β-estradiol (E2) was examined as a modulator of recombinant K_V_7.4 channels expressed in HEK293 cells. Whole-cell patch-clamp recordings showed that acute application of E2 reduced K_V_7.4 current density. At nanomolar concentrations, both E2 and the alternate GPER1 agonist aldosterone inhibited K_V_7.4 currents only when G protein-coupled estrogen receptor 1 (GPER1) was co-expressed, indicating a receptor-dependent mechanism. Pharmacological inhibition showed that phospholipase C (PLC) and protein kinase C (PKC), but not adenylyl cyclase, contribute to GPER1-mediated inhibition of K_V_7.4. In contrast, equivalent experiments performed with K_V_7.5 channels showed no significant effect of E2 despite GPER1 co-expression, suggesting subtype-selective regulation within the K_V_7 family. Surface biotinylation experiments suggest that GPER1 co-expression reduced surface K_V_7.4 expression, with a further reduction observed following E2 treatment. Finally, both E2 and aldosterone negatively regulate the sensitivity of K_V_7.2–5 activator ML213-mediated relaxation in arteries from female rats. Together, these findings identify K_V_7.4 as a downstream effector of rapid GPER1-dependent estrogen signaling and suggest that steroid status may influence K_V_7-targeted therapeutic responses.

## Introduction

K_V_7 (KCNQ) channels generate slowly activating, non-inactivating K^+^ currents that stabilize the membrane potential and limit cellular excitability [1–3]. Within the cardiovascular and visceral systems, K_V_7.4 channels are important for controlling smooth‑muscle tone and receptor-mediated responses, whereby their activity hyperpolarises smooth muscle, preventing Ca^2+^ entry, which drives relaxation. Their dysfunction has been implicated in hypertension, pulmonary vascular disease, and bladder overactivity [4–7].

K_V_7 channel function is modulated by sex steroid hormones and may contribute to sex- and cycle-dependent differences in cellular excitability and contractility [8,9]. More recently, rapid non-genomic estrogen signaling mediated by G-protein coupled estrogen receptor 1 (GPER1) has been implicated in the acute, short‑term regulation of ion channels and cellular excitability [10–12]. GPER1 is expressed in the sarcoplasmic reticulum (SR), Golgi apparatus, and plasma membrane of smooth muscle. GPER1 activation attenuates endothelin-1 responses and prevents angiotensinII (Ang-II)-mediated increases in blood pressure [13]. However, despite its largely vasodilatory role, GPER1 mediates protein kinase C (PKC)-dependent contraction in renal arteries, highlighting vascular bed-specific and context-dependent signaling [14].

Fluctuations in plasma 17β-estradiol (E2) during the estrous cycle are associated with altered arterial K_V_7.4 function, while activation of GPER1 reduces K_V_7.4 activity and membrane expression in rat arteries [9]. In the bladder, estrogen receptors including ERα and GPER1 regulate responses to K_V_7 activators, reinforcing the idea that steroid signaling converges on K_V_7 channels in multiple smooth‑muscle beds [15]. In both the heart and colonic epithelium, E2 inhibits K_V_7.1/KCNE1 via PKC-dependent mechanisms [8,16], indicating that estrogen-sensitive regulation of K_V_7 complexes can occur through acute kinase-dependent signaling. PKC-dependent channel attenuation has also been described for neuronal K_V_7/M-currents following Gq-coupled receptor activation [17,18].

Whilst E2-GPER1 signaling has been shown to reduce K_V_7.4 [9], the precise intracellular signaling cascade responsible for GPER1-mediated modulation of K_V_7.4 has yet to be fully defined. The present study investigates how E2 influences K_V_7.4 currents in HEK293 cells and whether these effects depend on GPER1 and specific G protein-coupled signaling cascades. By combining short‑term E2 incubation with pharmacological inhibitors of adenylyl cyclase, phospholipase C (PLC), and PKC, the experiments tested the hypothesis that GPER1 couples to Gq-PLC/PKC to mediate steroid-induced inhibition of K_V_7.4 currents. Clarifying this mechanism in an expression system provides a framework for understanding steroid regulation of K_V_7.4-mediated conductance in native smooth muscle and other tissues where multiple receptor pathways coexist.

## Materials and methods

### Cell culture and transfection

HEK293 cells stably expressing K_V_7.4 or K_V_7.5 were maintained in Dulbecco’s Modified Eagle Medium containing 4.5 g/L glucose and 4 mM L-glutamine, supplemented with 10% (vol/vol) fetal bovine serum and 1% penicillin/streptomycin. Cells were cultured at 37°C in a humidified atmosphere of 5% CO_2_ and passaged using standard techniques. For transient expression experiments, cells were transfected with a GPER1-GFP plasmid (0.5 µg) using Lipofectamine 2000 (Thermo Fisher Scientific) and recorded 24–48 h later.

### Electrophysiology

K_V_7.4 and K_V_7.5 currents were recorded using the whole-cell patch-clamp technique. The external solution contained (mmol/L): 140 NaCl, 4 KCl, 10 HEPES, 2 CaCl_2_, 1 MgCl_2_; the pH was adjusted to 7.3 with NaOH. Patch pipettes were pulled from borosilicate glass and had a resistance of 5–10 MΩ when filled with an internal solution containing (mmol/L): 30 KCl, 110 K-gluconate, 5 HEPES, 0.5 MgCl_2_, 0.5 EGTA, and 1 Na_2_-ATP.

Cells were voltage-clamped with a holding potential of −50 mV, and current amplitude was monitored by applying a 1 s test pulse to +40 mV at 15 s intervals. Current-voltage relationships were obtained using 1.5 s voltage steps from −70 mV to +40 mV in 10 mV increments, delivered every 15 s. Following each test pulse, membrane potential were stepped to −40 mV for 250 ms to record tail currents and then to −60 mV for 50 ms before returning to the holding potential of −50 mV. Currents were digitized and analyzed using Clampfit software; representative traces were generated by exporting data to text files and processing in GraphPad Prism.

### Drug application

E2 was prepared as stock solutions in ethanol and diluted into external solution immediately before use. For acute applications, drugs were applied via bath perfusion while continuously monitoring current responses at +40 mV. For incubation experiments, cells were exposed to 10 nM E2 for 30 min at 37°C before recording. The dependence of steroid effects on GPER1 was assessed by comparing responses in K_V_7.4-only cells with those in cells co-expressing GPER1-GFP.

### Signaling pathway inhibitors

To investigate downstream signaling, cells were preincubated with the adenylyl cyclase inhibitor SQ22536 (100 µM), the PLC inhibitor U73122 (1 µM), or the PKC inhibitors Chelerythrine (20 µM) and GF109203X (1 µM). Cells were first incubated with each drug alone for 5 min at 37°C, followed by addition of 10 nM E2 for a further 30 min at 37°C before recording. Current densities at +40 mV were determined under control, E2 alone, and combined drug and E2 treatment.

### Immunocytochemistry

HEK293 cells were seeded at approximately 50,000 cells onto 13 mm glass coverslips. Cells were transiently transfected with 0.5 µg KCNQ4-eGFP plasmid alone or co-transfected with 0.5 µg KCNQ4-eGFP and 0.5 µg GPER-Flag plasmids using polyethyleneimine (PEI). Following 48 h of expression, cells were treated with either vehicle control (100% ethanol) or 10 nM E2 for 30 min at 37°C. Cells were subsequently fixed with 4% paraformaldehyde (PFA) for 15 min at room temperature and washed three times with phosphate-buffered saline (PBS). Plasma membrane staining was performed using wheat germ agglutinin (WGA)-Texas Red for 10 min at room temperature, followed by three additional PBS washes. Coverslips were mounted onto glass slides using mounting medium containing DAPI for nuclear staining. Images were obtained with the confocal microscope A1R inverted (Nikon). NIS-Elements (version 6.10.02, Nikon) was used for image acquisition.

### Surface biotinylation

To investigate the effect of E2 on K_V_7.4 channel expression at the cell surface, HEK293 cells were co-transfected with 1 µg K_V_7.4 and 1 µg GPER1 or 1 µg EGFP and 24–48 h post-transfection, incubated with 10 nM E2 in culture media under standard culture conditions for 30 min. Post‑incubation, cells were washed 2× in cold PBS (pH 8), mechanically dislodged, pelleted, and resuspended in 0.5 mg/ml EZ-link Sulfo-NHS-SS biotin (ThermoFisher, UK) and incubated for 20 min with end-over-end rotation at 4°C. Cells were then quenched in 0.1 M glycine for 10 min. Following quenching, cells were centrifuged at 200 × g and lysed (25 mM Tris, 150 mM NaCl, 1% Triton X-100, and complete protease inhibitor cocktail (Roche; Merck, UK)) for 1 h at 4°C with end-over-end rotation. Lysates were clarified at 12,000 × g for 15 min at 4°C. Total protein content was determined by the BCA assay, as per the manufacturer’s instructions (Pierce, ThermoFisher, UK). 1 mg/ml cell lysate was added to 50 µl high-capacity streptavidin agarose (Pierce, ThermoFisher, UK) and incubated for 1 h at room temperature, followed by centrifugation at 2,000 × g for 5 min. The non-bound supernatant fractions were stored for later analysis. The agarose pellet was washed 4×, 5–10 min in lysis buffer, and then, biotinylated proteins were eluted in 50 µl 2× NuPAGE LDS +100 mM DTT.

Input and biotinylated fractions (bound; cell-surface) were separated on 4–12% NuPAGE gels (Invitrogen, ThermoFisher, UK) and transferred to nitrocellulose membranes using the iBlot system (ThermoFisher, UK). Membranes were blocked for 1 h with 5% nonfat milk in TBS-Tween 20 (0.1%; TBST). Primary antibodies, rabbit anti-GFP (1:5000, Genetex) for K_V_7.4-GFP and EGFP, and mouse anti-GAPH (1:1000, Antibodies.com), were incubated at 4°C overnight. Membranes were washed 3 times for 10–15 min with TBST and then incubated with either goat anti-rabbit IgG-HRP (1:7500; BioRad) or goat anti-mouse IgG-HRP (1:7500; BioRad) for 1 h at RT, followed by a further 3× TBST washes. SuperSignal West Pico PLUS chemiluminescent substrate (ThermoFisher, UK) was applied for signal detection (BioRad ChemiDoc imaging system).

### Animals

The animal experiments with Wistar Kyoto (WKY) rats were approved by the local Animal Care and Use Committees (institutional approval numbers P24-293). The experiments were conducted in accordance with the directives of the Danish National Committee on Animal Research Ethics and Danish legislation on experimental animals. Rats were 12–15 weeks of age and were euthanized by cervical dislocation following a percussive blow to the head. Estrus cycle state determination was performed as per [9]. In short, ice-cold dissecting physiological salt solution containing (HEPES PSS, in mM): 120 NaCl, 6 KCl, 1.2 MgCl_2_, 12 glucose, 10 HEPES, with pH balanced at 7.4 via NaOH, was flushed inside the vaginal canal 3×. The subsequent cell suspension was plated and imaged. The presence and absence of small nucleated epithelial cells, large anucleated keratinized epithelial cells, and neutrophils allowed for stage differentiation, by reference to [19].

### Wire myography

Segments of third‑order mesenteric arteries, ~2 mm in length, were dissected of fat and peripheral tissue in ice-cold dissecting HEPES PSS. Arteries were mounted on two 40 μm steel wires within a wire myograph chamber (Danish Myo Technology, Aarhus, Denmark) filled with physiological salt solution (PSS, 5 mL), containing (in mM): 121 NaCl, 2.8 KCl, 1.6 CaCl_2_, 25 NaHCO_3_, 1.2 KH_2_PO_4_, 1.2 MgSO_4_, 0.03 EDTA, and 5.5 glucose, oxygenated with 95% O_2_ and 5% CO_2_ at 37°C. Signals were digitized via PowerLab and recorded via LabChart (AD Instruments).

Arteries were left to warm for 30 min, then normalized to an internal luminal circumference equal to that seen at a 90% of a transluminal pressure of 100 mmHg (13.3 kPa) [20]. Subsequently, vessels underwent an equilibration protocol, whereby they were left for 10 min, then contracted with a high K^+^ physiological salt solution (KPSS) of the following composition (in mmol-L-1): 63.5 NaCl, 60 KCl, 1.6 CaCl_2_, 25 NaHCO_3_, 1.2 KH_2_PO_4_, 1.2 MgSO_4_, 0.03 EDTA, and 5.5 glucose to assess viability. Constriction in response to KPSS was allowed to plateau before washing the vessel in PSS (≥4x). This step was repeated, after which endothelial cell integrity was determined by pre-constricting the vessel with 10 μmol-L-1 α-1 adrenoreceptor agonist methoxamine, then relaxing the vessel by the addition of 10 μmol-L-1 acetylcholine mimetic carbachol. A relaxation of >90% was deemed as endothelial cell positive.

After equilibration, vessels were preincubated in either DMSO solvent control, E2 (10 nM), or aldosterone (10 nM) for 30 min prior to pre-constriction with methoxamine (10 μM). After stable tone was achieved, concentration-effect curves were generated in half-log steps in response to ML213 (0.01–10 μM).

### Data analysis and statistics

Current density was calculated by normalizing current to cell capacitance, and current-voltage relationships were constructed from the steady-state current at the end of each pulse. Activation kinetics were assessed from the first 400 ms of each trace. Statistical analyses were performed using GraphPad Prism; data normality was assessed with the Shapiro–Wilk test, and parametric Student’s t-tests (paired or unpaired with Welch’s correction) or two-way ANOVA with false discovery rate control were applied as appropriate. All concentration effect curves are displayed as log‑transformed values (X = LogX) with a non-linear regression analysis via the following equation: Y = Bottom + (Top-Bottom)/(1 + 10^((LogEC50-X)*HillSlope)). Data were analyzed via GraphPad Prism. Data are presented as mean ± SEM, and *n* indicates the number of cells or animals.

## Results

### Acute E2 application inhibits K_V_7.4 currents

**Figure 1.**
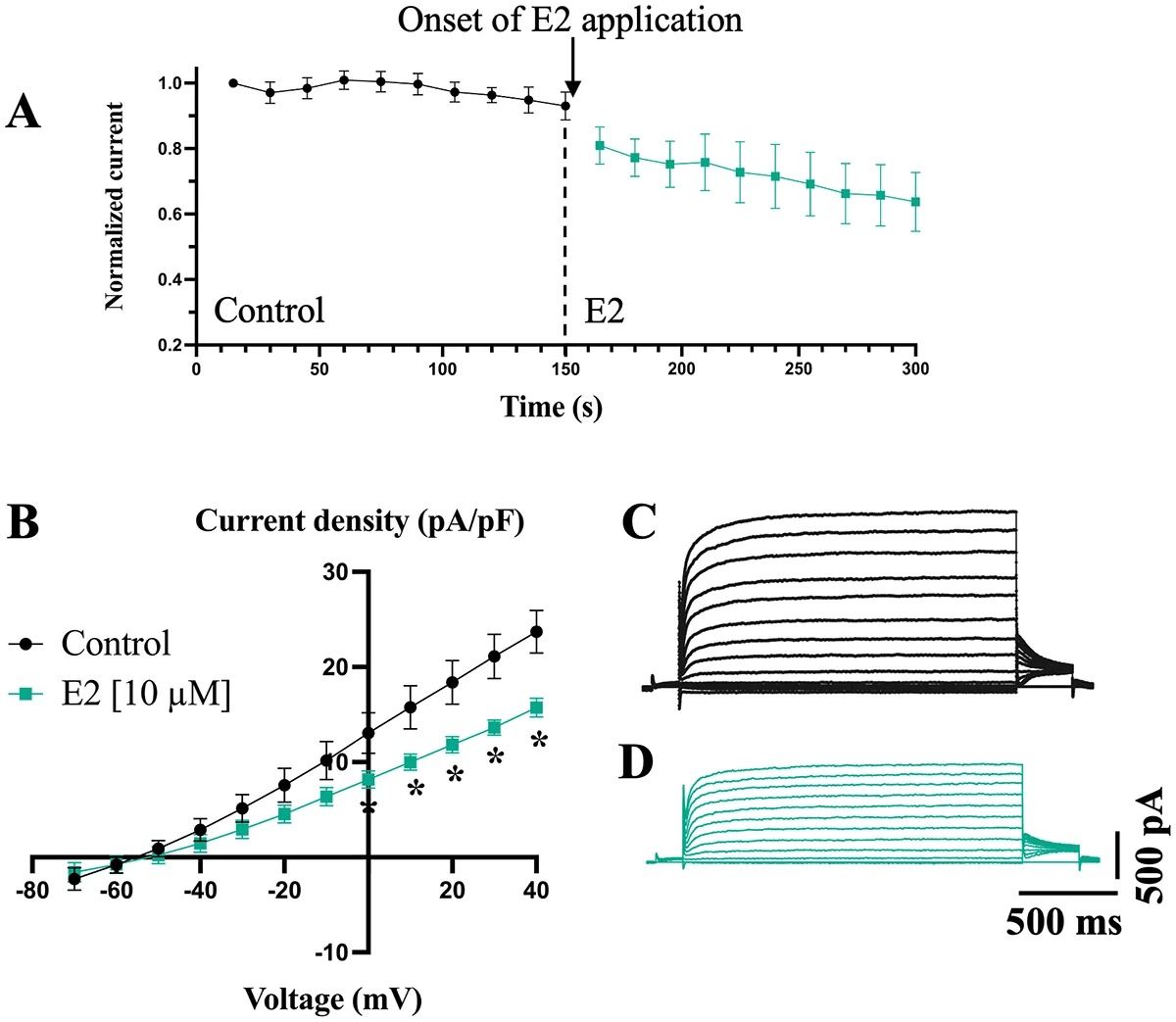
Acute E2 application decreases K_V_7.4 current. (A) Time course of normalized current at +40 mV during acute application of 10 µM E2 and (B) current-voltage relationships before and after drug application in HEK293 cells expressing K_V_7.4. (C–D) representative traces illustrating reduced current (*n* = 6).

Acute application of 10 µM E2 produced a progressive reduction in K_V_7.4 current amplitude measured at +40 mV, independent of GPER1 expression. The relationship between time and normalized current revealed a progressive decline in current following E2 exposure, which stabilized at a reduced steady-state level (Figure 1A).

Current-voltage relationships constructed from steady-state currents at the end of 1.5 s pulses showed that 10 µM E2 significantly reduced K_V_7.4 current density at voltages ≥ 0 mV (Figure 1B). At +40 mV, current density fell from 23.7 ± 2.2 pA/pF under control conditions to 15.7 ± 0.9 pA/pF after 10 µM E2 (*n* = 6; *p* ≤ 0.05 versus control). Representative current traces confirmed substantial inhibition following E2 application (Figure 1C,D).

### GPER1 enables steroid sensitivity at nanomolar concentrations

**Figure 2.**
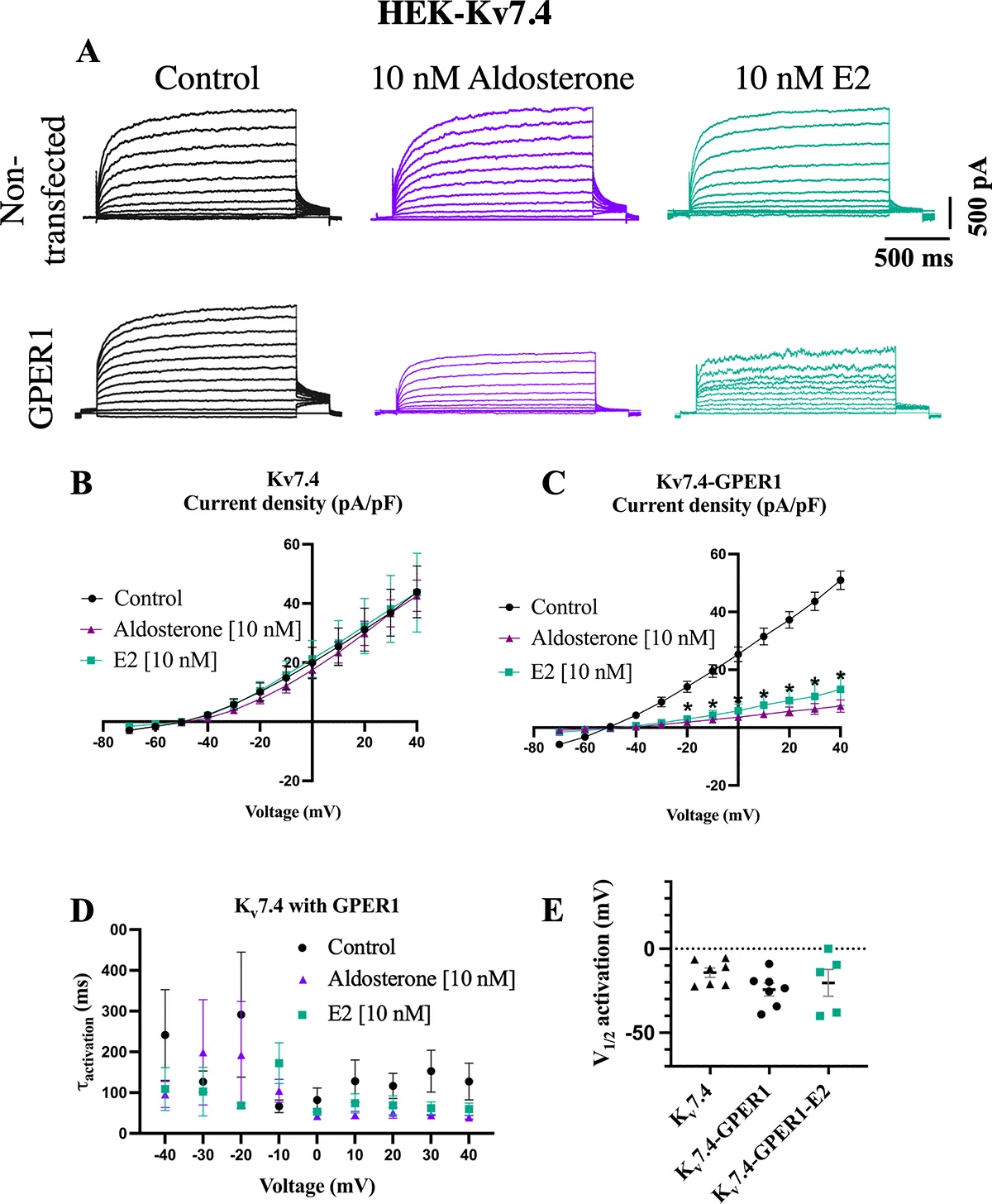
Nanomolar concentrations of E2 inhibit K_V_7.4 currents only in the presence of GPER1. (A) Whole-cell current traces recorded from HEK293 cells expressing K_V_7.4 alone (above), or co-expression GPER1 (below) under control conditions and following incubation with 10 nM Aldosterone and E2. (B) Current-voltage relationships showing no significant effect of 10 nM E2 on Kv7.4 current density in the absence of GPER1 co-expression. (C) Current-voltage relationships from HEK293 cells co-expressing K_V_7.4 and GPER1, demonstrating significant inhibition of K_V_7.4 current density following incubation with 10 nM E2. (D) Analysis of activation kinetics during the first 400 ms of depolarization revealed no significant changes following treatment with E2 or aldosterone. Data are presented as mean ± SEM; *n* = 5 cells per group. Statistical comparisons were performed using Student’s t-test. (E) Voltage-dependence of activation for K_V_7.4 currents. Data are presented as mean ± SEM with individual cells shown (*n* = 5–7 cells per group). Statistical analysis was performed using one-way ANOVA (*p* > 0.05).

E2 at physiologically-relevant, nanomolar concentrations, did not significantly affect K_V_7.4 current density in cells expressing K_V_7.4 alone (Figure 2A,B) but co-expression of GPER1 rendered K_V_7.4 currents sensitive to E2 (Figure 2A-C). In K_V_7.4/GPER1 co-expressing cells, 10 nM E2 reduced current density significantly (*n* = 5; *p* < 0.05), with representative traces illustrating substantial inhibition across the voltage range tested. Analysis of the activation kinetics during the first 400 ms of each trace revealed no significant changes in the presence of E2 (Figure 2D), indicating that E2 did not produce obvious alterations in the activation profile under these recording conditions. Similarly, V_1/2_ activation values derived from Boltzmann fits were not significantly different between the K_V_7.4, K_V_7.4-GPER1, and K_V_7.4-GPER1-E2 groups (one-way ANOVA, *p* > 0.05; Figure 2E). Aldosterone also activates GPER1 in addition to conventional mineralocorticoid receptors [21]. Application of 10 nM aldosterone also reduced K_V_7.4 currents in a GPER1-dependent manner, and activation kinetics were not affected (Figure 2B-D).

**Figure 3.**
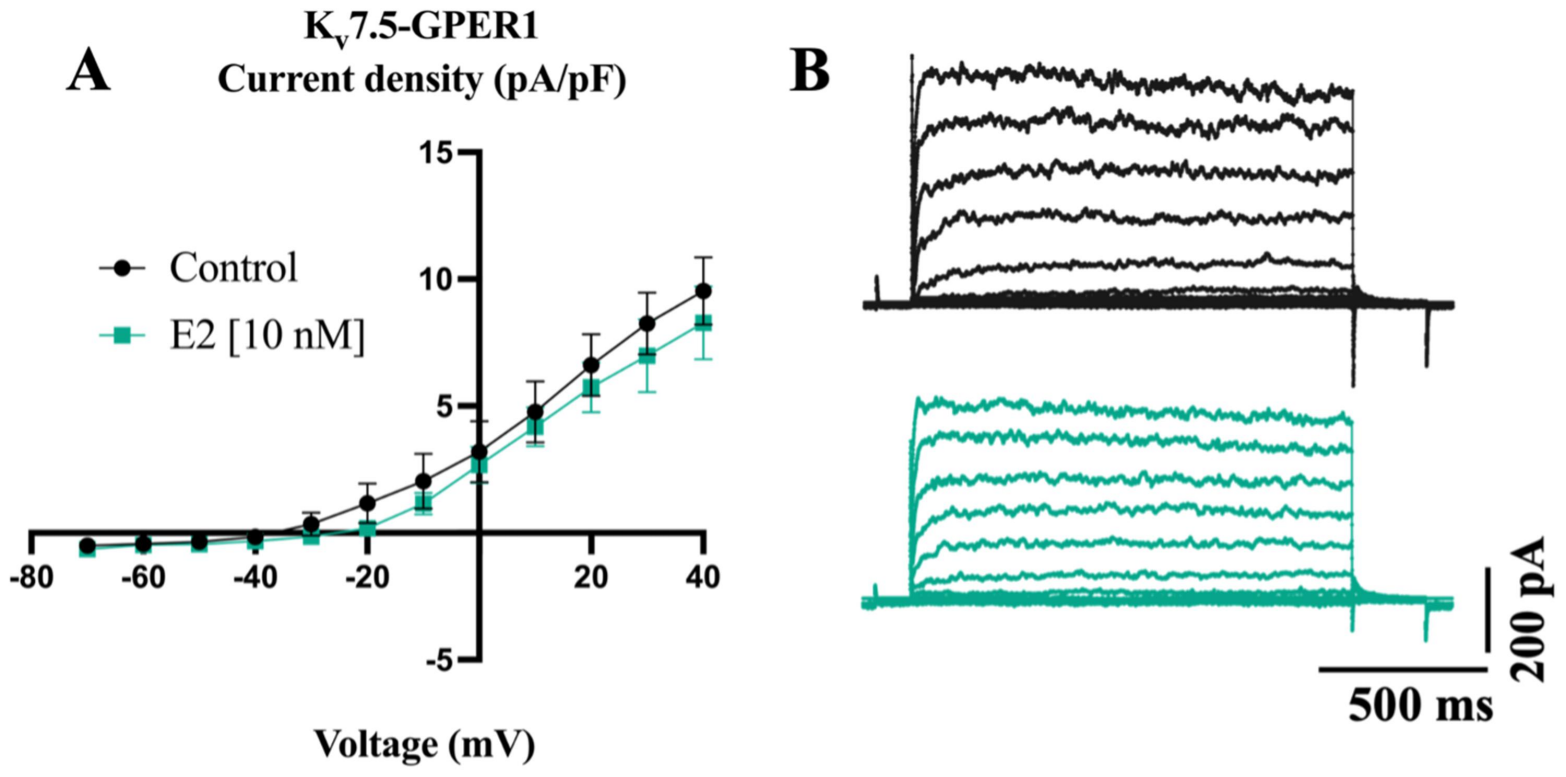
K_V_7.5 currents are not significantly altered by E2 in GPER1-expressing HEK293 cells. (A) Current-voltage relationships for currents measured at the end of 1.5 s pulses before and after treatment with 10 nM E2 in HEK293 cells stably expressing K_V_7.5 and transiently transfected with GPER1. Data are presented as mean ± SEM (*n* = 5). (B) Representative current recordings of control (black) and E2-treated (green) cells.

A series of experiments were undertaken to ascertain if GPER1 activation regulated the closely related channel K_V_7.5. In contrast to K_V_7.4, incubation with 10 nM E2 did not significantly alter K_V_7.5 current density in GPER1‑expressing cells (current density at +40 mV: control, 9.5 ± 1.3 pA/pF; 10 nM E2, 8.2 ± 1.4 pA/pF; Figure 3A,B). Current-voltage relationships were comparable between control and E2-treated cells (Figure 3A,B), indicating that acute GPER1-dependent inhibition was not evident for K_V_7.5 under these experimental conditions.

### PLC and PKC signaling contribute to GPER1-dependent K_V_7.4 inhibition

**Figure 4.**
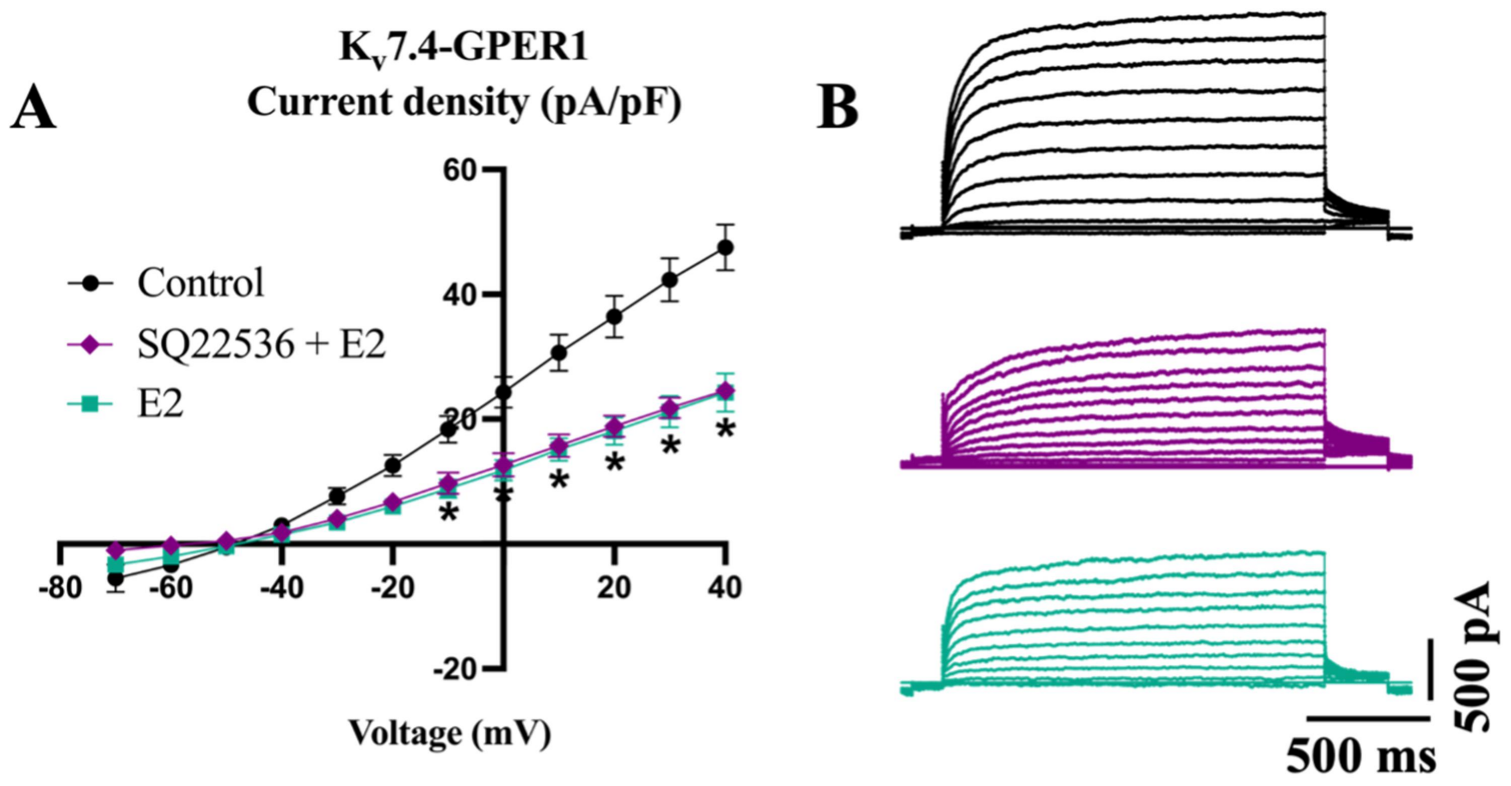
Inhibition of adenylyl cyclase does not prevent the reduction of K_V_7.4 currents by E2. (A) Current-voltage relationships for currents measured at the end of 1.5 s pulses before and after 10 nM E2 and 100 µM SQ22536 (adenylyl cyclase inhibitor) plus 10 nM E2 for 30 min at 37°C on HEK293 cells co-expressing K_V_7.4 and GPER1. Data are presented as mean ± SEM (*n* = 6/group). (B) Representative current recordings of control (black), SQ22536 + E2 (purple), and E2 (green).

GPER1 is a G‑protein-coupled receptor that can elicit signals through Gαq and Gαs leading to increased phospholipase C and adenylyl cyclase, respectively [22]. To ascertain the signaling pathways underlying GPER1-dependent inhibition of K_V_7.4, we used specific inhibitors of signaling checkpoints. Incubation with 10 nM E2 significantly reduced current density in the presence of the adenylyl cyclase inhibitor SQ22536, with current-voltage relationships remaining attenuated relative to control conditions (Figure 4A,B). At +40 mV, current density was 47.5 ± 3.6 pA/pF in control cells, 24.6 ± 1.6 pA/pF following treatment with 10 nM E2, and 24.2 ± 3.0 pA/pF in cells treated with 100 µM SQ22536 plus 10 nM E2 (*n* = 6; *p* < 0.05 versus control; Figure 4A,B). These observations suggest that adenylyl cyclase signaling does not contribute substantially to E2-induced inhibition of K_V_7.4 currents.

**Figure 5.**
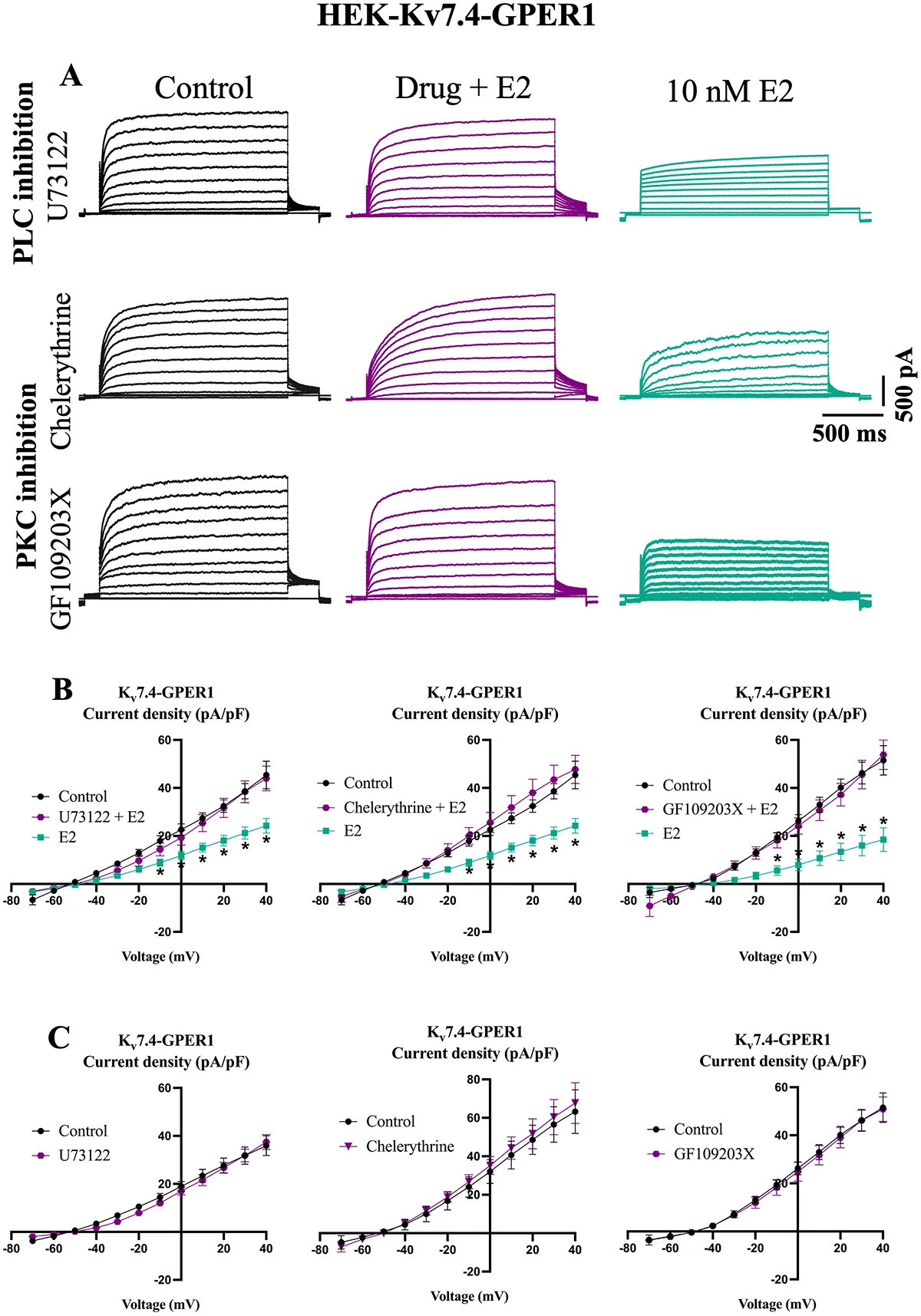
PLC and PKC inhibition prevent E2-induced attenuation of K_V_7.4 currents in GPER1-expressing cells. (A) Representative whole-cell current traces recorded from HEK293 cells co-expressing K_V_7.4 and GPER1 under control conditions, after incubation with 10 nM E2, and following treatment with pathway inhibitors in the presence of E2. Current-voltage relationships obtained from cells treated with (B) pathway inhibitors in combination with E2 and (C) each inhibitor alone. Data are presented as mean ± SEM (*n* = 5–6 cells per group).

In contrast, inhibition of PLC with U73122 prevented the reduction of K_V_7.4 currents by E2. In cells treated with U73122 (1 µM) plus E2, K_V_7.4 current density at +40 mV (43.9 ± 5.1 pA/pF) was comparable to control values (45.5 ± 5.7 pA/pF), indicating that PLC inhibition abolished the E2-induced reduction in current density (*n* = 6; Figure 5A,B).

Similarly, blockade of PKC with either Chelerythrine (20 µM) or GF109203X (1 µM) prevented the inhibitory effect of E2. In the presence of Chelerythrine, current density at +40 mV remained at 47.7 ± 5.8 pA/pF compared with 45.4 ± 5.7 pA/pF in control cells (*n* = 6), while GF109203X-treated cells exhibited current densities of 53.8 ± 6.1 pA/pF versus 51.5 ± 6.1 pA/pF in controls (*n* = 5; Figure 5A,B). Importantly, neither U73122, Chelerythrine, nor GF109203Xaffects K_V_7.4 currents when incubated alone (*n* = 5; Figure 5C).

### Membrane localization of K_V_7.4 after GPER1 activation

**Figure 6.**
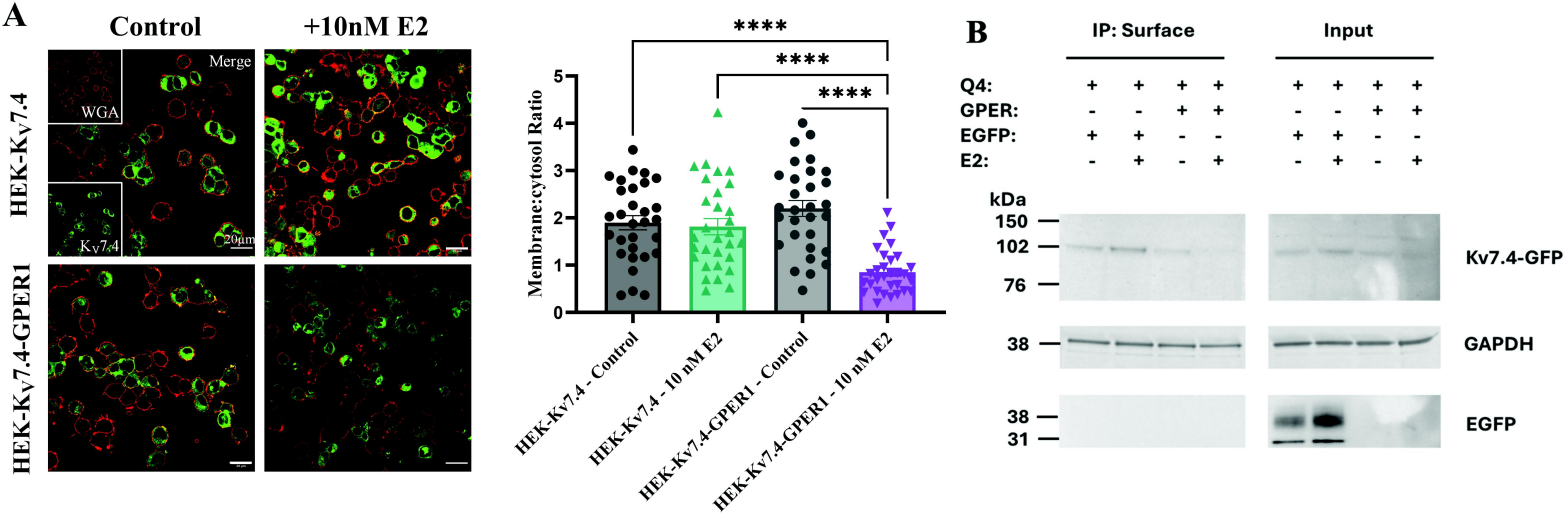
GPER1 and E2 reduce surface expression of K_V_7.4 channels in HEK293 cells. (A) Representative confocal images of HEK293 cells expressing K_V_7.4-GFP under control conditions or following treatment with 10 nM E2 in the absence or presence of GPER1 expression. K_V_7.4-GFP fluorescence is shown in green and membrane staining in red. Summary quantification of membrane fluorescence intensity is shown on the right. Data are presented as mean ± SEM, with individual cells displayed as scatter points (*n* = 20–30). Statistical significance was assessed via ordinary one-way ANOVA with Bonferroni post hoc testing (*p ≤ 0.05, **p ≤ 0.01). (B) Representative surface biotinylation assay showing surface and input fractions from HEK293 cells expressing K_V_7.4-GFP in the presence or absence of GPER1 and E2 (10 nM). GAPDH was used as a loading control for both surface and input fractions. EGFP signal was detected only in input samples and not in surface fractions, indicating minimal carry-over during streptavidin isolation.

Experiments were performed to ascertain if the reduction in K_V_7.4 channel currents was due to a reduction in K_V_7.4 protein from the cell membrane as previously demonstrated in isolated rat arterial smooth muscle cells [9]. Immunocytochemistry showed that GFP-tagged K_V_7.4 resided predominantly in the cell membrane (Figure 6A). K_V_7.4 membrane expression was reduced by co-expression with GPER1 and further reduced by subsequent treatment with 10 nM E2 for 30 min (Figure 6A). See Supplemental Figure 1 for all images used within the analysis.

Qualitative surface biotinylation confirmed that GPER1 reduced K_V_7.4 membrane abundance again with a further decrease upon 10 nM E2 treatment. GAPDH signal intensity was similar across input samples, supporting consistent protein loading (Figure 6B). Similarly, GAPDH was also detected at equivalent abundance within surface fractions, which has previously been reported in membrane-associated preparations [23]. EGFP signal was restricted to input samples and was not observed in surface fractions, suggesting minimal carry-over during the streptavidin isolation of biotinylated proteins. These observations are consistent with the electrophysiological data and support the possibility that GPER1 signaling alters K_V_7.4 surface expression. See Supplemental Figure 2 for the full, non-cropped blot used in Figure 6(B).

### E2 and aldosterone impair K_V_7-mediated relaxations in whole arteries

**Figure 7.**
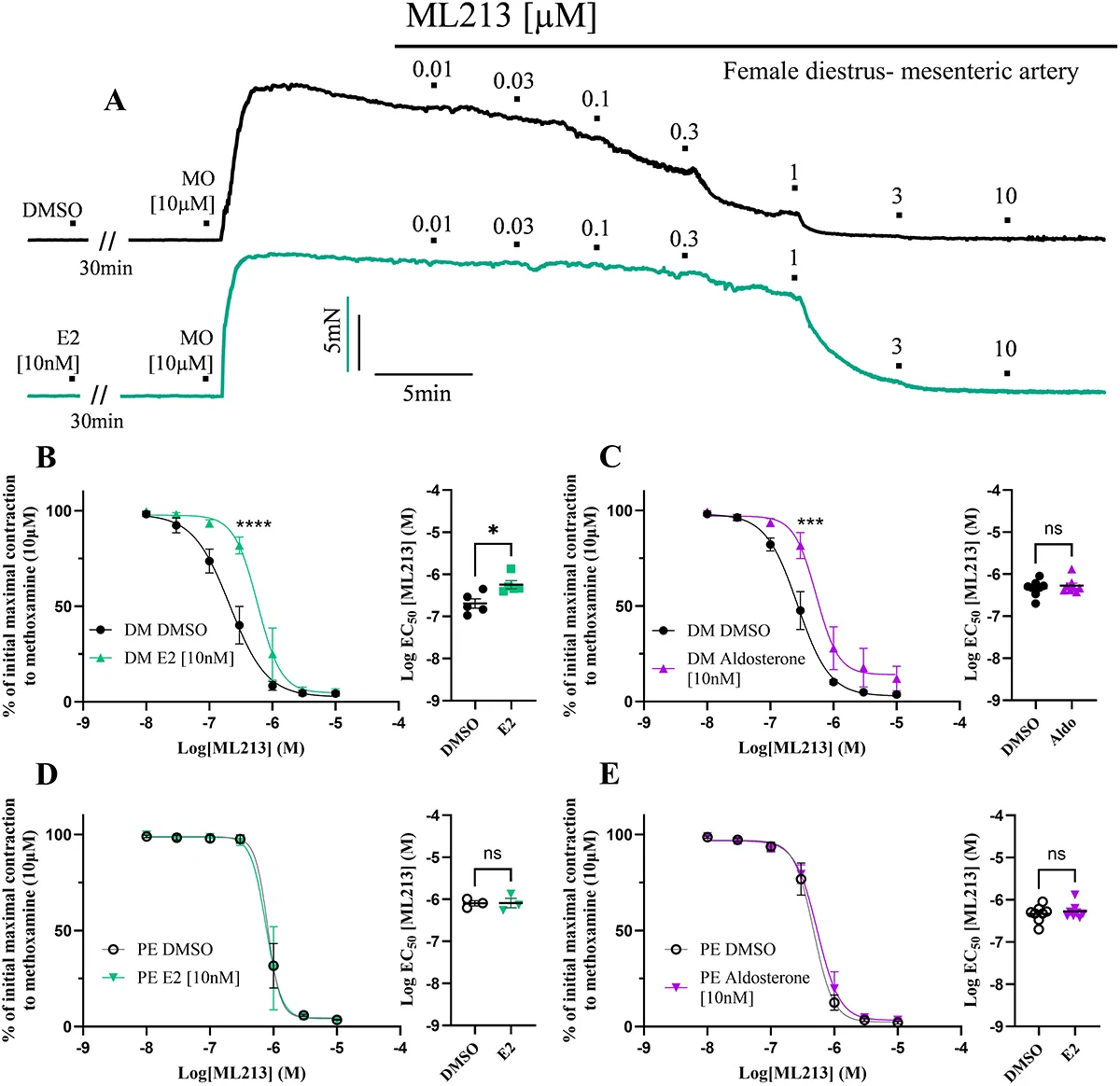
E2 and aldosterone impair relaxations to K_V_7.2–7.5 activator in whole arteries. (A) Representative trace of, and (B) mean data and EC_50s_for K_V_7.2–5 activator ML213 (0.01–10 µM) mediated relaxation of pre-constricted arterial tone (10 µM methoxamine) in mesenteric arteries harvested from a female Wistar Kyoto rat during diestrus/metestrus (D/M), pre-incubated for 30 min either with DMSO solvent control (black) or estradiol E2 (E2; green; 10 nM; *n* = 5). (C) Mean data for ML213‑mediated relaxation for vessels incubated in either DMSO or aldosterone (10 nM; *n* = 7). The effect of E2 (D) and aldosterone (E) on ML213‑mediated in mesenteric arteries harvested from rats during pro-oestrus and oestrus (P/E, *n* = 3–7). All data are expressed as mean ±SEM. (*n* =) number of animals used. Statistical analysis included a 2-way ANOVA with a post hoc Sidak’s correction or a Student’s t-test; * = *P*0.05, *** = *P*0.001, and **** = *P*0.0001.

A final series of experiments was performed on mesenteric arteries from female rats across the estrus cycle, separated into diestrus and metestrus (D/M), and pro-estrus and estrus (P/E). These groupsrepresent the low and high serum E2 stages of the cycle, respectively [9]. The K_V_7.2–7.5 activator ML213 [24] relaxed arteries precontracted with methoxamine (10 µM) in a concentration-dependent manner (Figure 7A). ML213‑mediated relaxation was impaired by preincubation of the artery in both 10 nM E2 and 10 nM aldosterone for 30-min prior to contraction in arteries from females during D/M (Figure 7B,C). Comparably, neither E2 nor aldosterone affected ML213‑mediated relaxation in arteries harvested from animals during P/E, where serum E2 is higher (Figure 7D,E). These data show that GPER1 regulation of K_V_7.4 is physiologically relevant.

## Discussion

This study demonstrates that physiological concentrations of E2 inhibit K_V_7.4 currents via a GPER1-dependent Gq-PLC/PKC pathway in HEK293 cells. Micromolar concentrations of E2 directly reduced K_V_7.4 currents independently of GPER1 expression, consistent with a receptor-independent mechanism, potentially through steric hindrance of channel function via a direct channel block as previously described for K_V_ channels [25]. In contrast, nanomolar concentrations of E2 required GPER1 expression, indicating that membrane-initiated receptor signaling is particularly relevant at physiologically relevant hormone levels. Although lower E2 concentrations were not experimentally assessed, these findings suggest that endogenous-range E2 concentrations act predominantly through GPER1-dependent signaling pathways. The absence of major changes in the kinetics or voltage-dependence of activation suggests that this signaling primarily reduces maximal conductance or channel availability, consistent with PKC-dependent modulation reported for other K_V_7 complexes [26]. These findings are supported by data in native smooth muscle cells, whereby E2 and aldosterone impair sensitivity to K_V_7.2–5 activator‑mediated relaxation in rat mesenteric arteries.

This study complements and extends previous work showing that estrous cycle-dependent changes in arterial K_V_7.4 function are mediated in part by GPER1 signaling [9], which reduces K_V_7.4 function and surface expression and promotes a pro-contractile phenotype in the artery. By isolating recombinant K_V_7.4 and GPER1 in HEK293 cells, the present data identify Gq-PLC/PKC as a sufficient pathway to inhibit K_V_7.4 current, independent of the complex milieu of native vascular or bladder tissues. The observation that PKC inhibition abrogated E2-induced suppression of K_V_7 currents/membrane abundance also aligns with reports that E2 inhibits both cardiac K_V_7.1/KCNE1 function [8] and colonic epithelial K_V_7.1/KCNE3 function and subcellular trafficking [16,27], suggesting that PKC-dependent mechanisms may represent a common strategy through which estrogenic signals regulate distinct K_V_7 channel complexes [28]. Because K_V_7 channels are highly dependent on membrane phosphatidylinositol 4,5-bisphosphate (PIP_2_) [29–31], PLC activation may additionally reduce K_V_7.4 currents through depletion of PIP_2_ availability.

An additional finding of this study was that GPER1-dependent inhibition was observed for K_V_7.4 but not K_V_7.5 channels. Although K_V_7.4 and K_V_7.5 share substantial sequence homology, the absence of a detectable effect in K_V_7.5 suggests that subtype-specific structural determinants may contribute to E2 sensitivity. Interestingly, there is no strong candidate for a classical PKC consensus site (**S/T–x–R/K**) predicted for K_V_7.4, whilethere are in K_V_7.5. However, there are pseudo consensus sites (RLYSTDMSRAYLTAT at position 358 and SPTKVQKSWSFNDRT at 482) that are unique to K_V_7.4. Given that pharmacological inhibition of PLC and PKC prevented E2-dependent inhibition of K_V_7.4 currents, differential phosphorylation sensitivity may contribute to the selective regulation of K_V_7.4. However, the present data do not establish direct phosphorylation of this site, and further studies using mutagenesis and phospho-specific approaches will be required to determine its functional significance.

Some debate within the literature argues as to the relationship between aldosterone, the mineralocorticoid receptor, and GPER1 signaling [32]. More recently, expression of GPER1 within insect Sf9 cells demonstrates that, in the absence of mammalian intracellular machinery, particularly the mineralocorticoid receptor, aldosterone binds to GPER1 at a greater potency than E2 [33]. Our work here similarly demonstrates that, in both heterologous overexpression systems and in native smooth muscle, the inhibitory effects of both aldosterone and E2 on K_V_7.4 currents and function, respectively, are analogous. Supporting the notion of direct aldosterone-GPER1 signaling, and that independent of which agonist activates the receptor, K_V_7.4 remains a downstream target. Further, our data demonstrate that this pathway can be saturated by natural fluctuations of sex-hormones across the estrous cycle, as neither E2 nor aldosterone impaired ML213‑mediated relaxation in arteries from animals where serum E2 is already raised [9].

The identification of discrete nodes within the GPER1-K_V_7.4 pathway has potential translational implications. Pharmacological modulation of GPER1, PLC, or PKC may alter the steroid sensitivity of K_V_7.4 in conditions where altered estrogen levels contribute to vascular, pulmonary, or bladder dysfunction. Conversely, K_V_7 channel activators developed for cardiovascular or urogenital indications may need to be evaluated under conditions of variable steroid status and GPER1 activity. Future studies should test whether similar GPER1-Gq-PLC/PKC mechanisms operate in native tissues and in vivo, and whether chronic hormone exposure engages additional transcriptional and trafficking pathways that modify K_V_7.4 expression and localization over longer timescales.

## Supplementary Material

Supplementary material.docx

## Data Availability

The data supporting the findings of this study are available from the corresponding author upon reasonable request.
