# Supplementary material for: Physiological 17β-oestradiol inhibits KV7.4 currents via GPER1-dependent PLC/PKC signalling
Source: Channels (Austin). 2026 Sep 10;20(1):2729646. doi: 10.1080/19336950.2026.2729646 (PMC13568567; doi:10.1080/19336950.2026.2729646)
Supplement: Supplementary file 1 [file KCHL_A_2729646_SM8906.docx]

**Supplementary material**

**Supplemental figure 1. Uncropped surface biotinylation western blots.** Upper panel: surface and input fractions probed with anti-GFP, detecting the GFP-tagged Kv7.4 and EGFP from the transfection control (to ensure equivalent total cDNA across all samples). Lower panel: the lower half of the membrane (cut between 38 and 52 kDa) was probed with anti-GAPDH for loading control.

**Supplemental figure 2. GFP-tagged K_V_7.4 expression in HEK293 cells.** Confocal images of all HEK293 cells used in analysis described in figure 6. Images show HEK293 expressing Kv7.4-GFP under control conditions (left) or following treatment with 10 nM E2 (right) with (above) or without (below) co-expression with GPER1. Kv7.4-GFP fluorescence is shown in green and membrane staining in red.
